# Supplementary material for: Inverse design of 3D reconfigurable curvilinear modular origami structures using geometric and topological reconstructions
Source: Nat Commun. 2022 Dec 3;13:7474. doi: 10.1038/s41467-022-35224-2 (PMC9719498; doi:10.1038/s41467-022-35224-2)
Supplement: Supplementary file 1 — Supplementary Information [file 41467_2022_35224_MOESM1_ESM.pdf]

Supplementary Information

Inverse design of 3D reconfigurable curvilinear modular origami structures using geometric and topological reconstructions

Kai Xiao[1], Zihe Liang[1], Bihui Zou[1], Xiang Zhou[2*], Jaehyung Ju[1*]

1. UM-SJTU Joint Institute, Shanghai Jiao Tong University, 800 Dongchuan Road, Shanghai, China

2. School of Aeronautics and Astronautics, Shanghai Jiao Tong University, 800 Dongchuan Road, Shanghai, China

Supplementary Note 1: Geometric reconstruction

Construction of 2D modular origami structures

Our geometric reconstruction primarily used for 3D curvilinear architected structures in the main text can also be applied to planar cases, as illustrated in Supplementary Figure 1. A unit cell can be mapped to a target planar geometry followed by planar shrinkage. One can extrude tubes parallel to the lines connecting adjacent centroids of the shrunken polyhedrons to make origami modules, connecting with adjacent modules to construct an assembly.

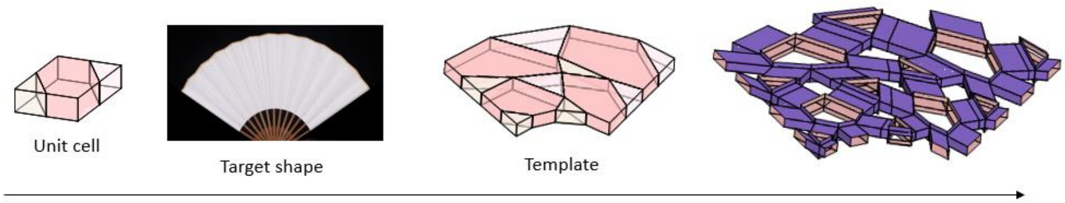

Supplementary Figure 1. Geometric reconstruction of a planar 2D curvilinear architected structure with a $2 \times 2 \times 1$ *mapping of a unit cell.*

Size dependency on mapping

We quantify the effect of mapping density on the volumetric change of a unit cell. We fill a sphere by mapping a cubic unit cell with different system sizes, as shown in Supplementary Figure 2a. The selected polyhedron, which is commonly close to the boundary of the template, undergoes a severe volume change as the system size increases, as illustrated in Supplementary Figure 2b. The volume change $\left(V_{p_0} - V_p\right)/V_{p_0}$ of the polyhedron continuously increases from 49.9% to 84.3% as the mapping density increases from $2 \times 2 \times 2$ to $6 \times 6 \times 6$, where V_{p_0} and V_p are the volumes of the polyhedron before and after mapping, respectively.

We also quantify the effect of the mapping density on the filling efficiency, as indicated by the volumetric ratio V_g/V_0 in Supplementary Figure 2c. V_g and V_0 are the volumes of the template and the target shape, respectively. Although it is apparent that a denser mapping approximates the targeting shape with higher accuracy, we notice a specific system size may balance the accuracy and computational cost. In this example with a spherical target shape, a $3 \times 3 \times 3$ system provides a good filling accuracy of V_g/V_0 $(= 90.62\%)$, close to the filling accuracy of a $4 \times 4 \times 4$ system with V_g/V_0 $(= 93.76\%)$. Note that the $3 \times 3 \times 3$ system uses a much smaller number of polyhedrons (27) than the $4 \times 4 \times 4$ system (64),

indicating the filling efficiency of the $3 \times 3 \times 3$ system.

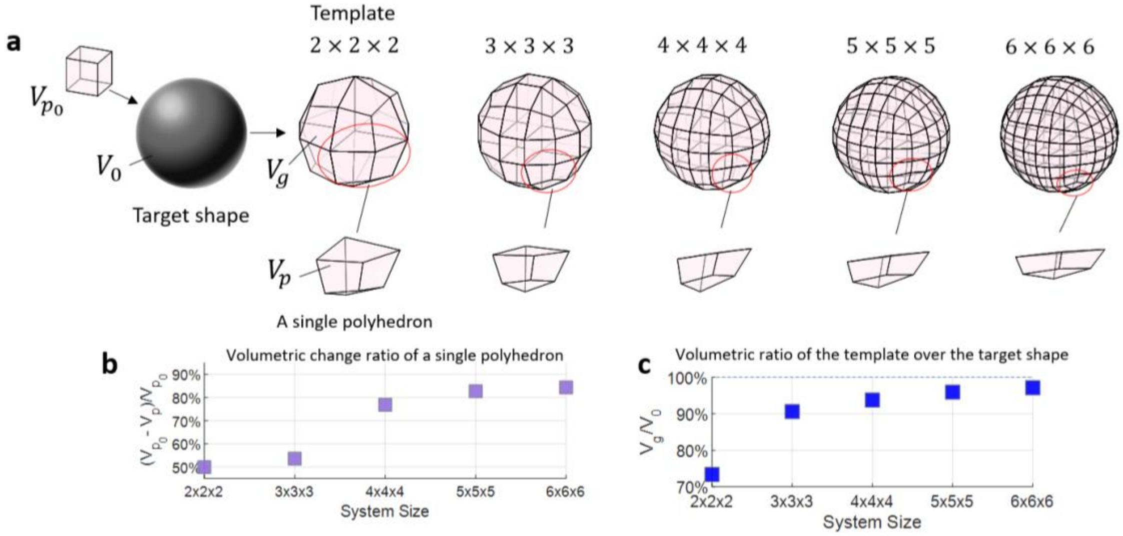

Supplementary Figure 2. Effect of system size on the volume of mapping template: (a) mapping of cubic unit cells into spheres with different system sizes; a single polyhedron at the same corner of the template with a different system size is shown to compare mapping densitie; (b) volumetric change ratio $\left(V_{p_0} - V_p\right)/V_{p_0}$ of a single polyhedron with mapping size; (c) effect of denser mapping on the ratio V_g/V_0 , where V_g is the template volume and V_0 is the volume of the target shape.

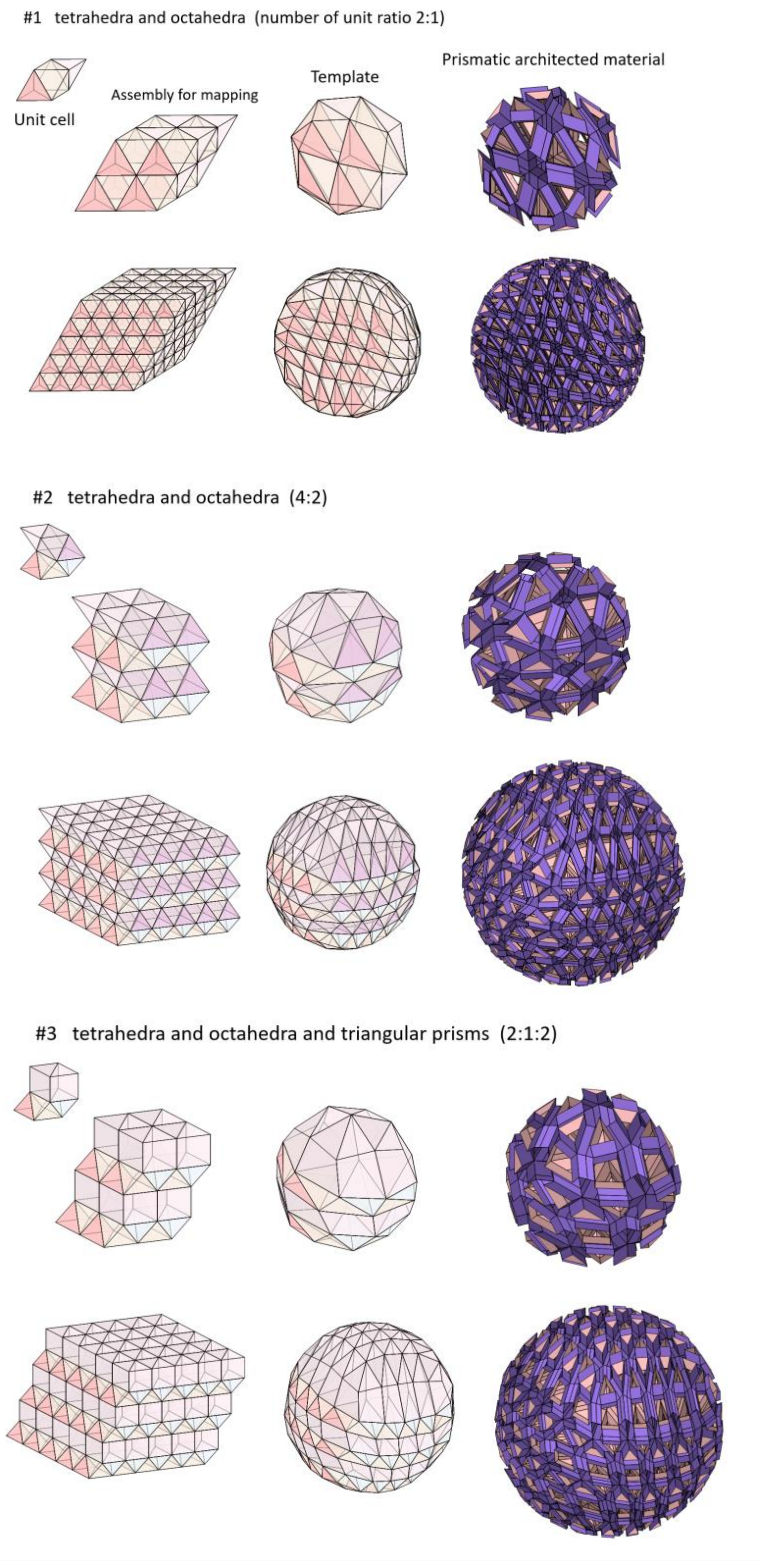
#1 tetrahedra and octahedra (number of unit ratio 2:1)
Unit cell
Assembly for mapping
Template
Prismatic architected material
#2 tetrahedra and octahedra (4:2)
#3 tetrahedra and octahedra and triangular prisms (2:1:2)

#4 tetrahedra and octahedra and triangular prisms (4:2:4)

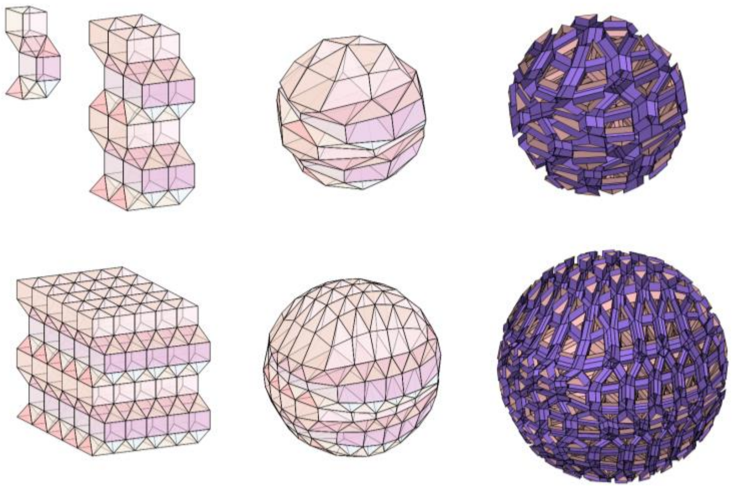

#5 tetrahedra, rhombicuboctahedra and cubes (2:1:1)

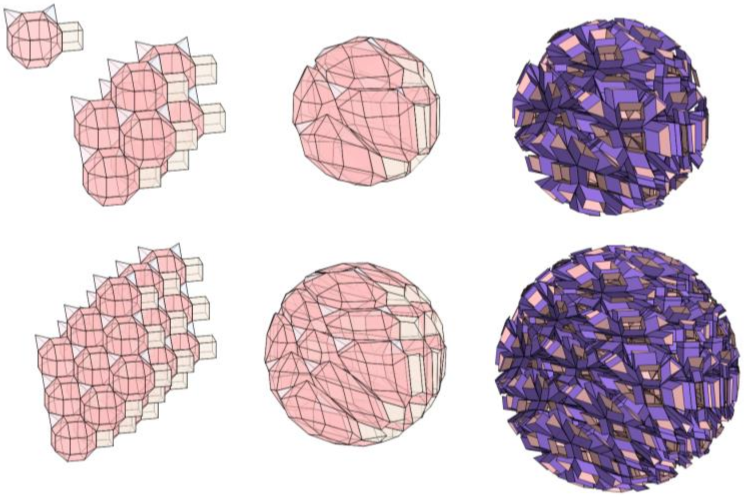

#6 tetrahedra and truncated tetrahedra (2:2)

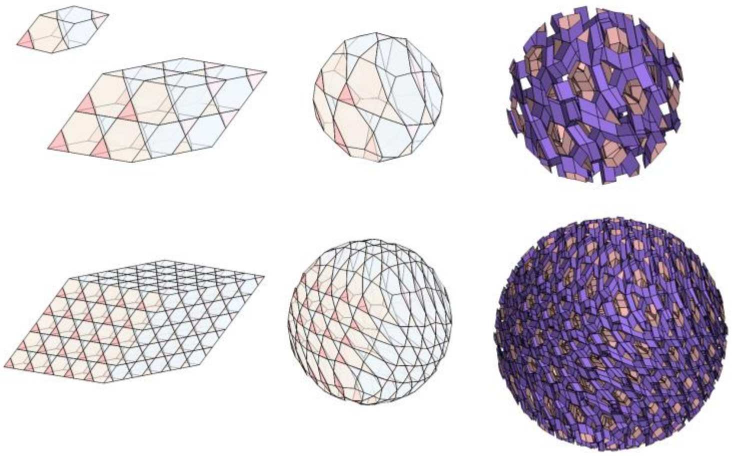

#7 octahedra and cuboctahedra (1:1)

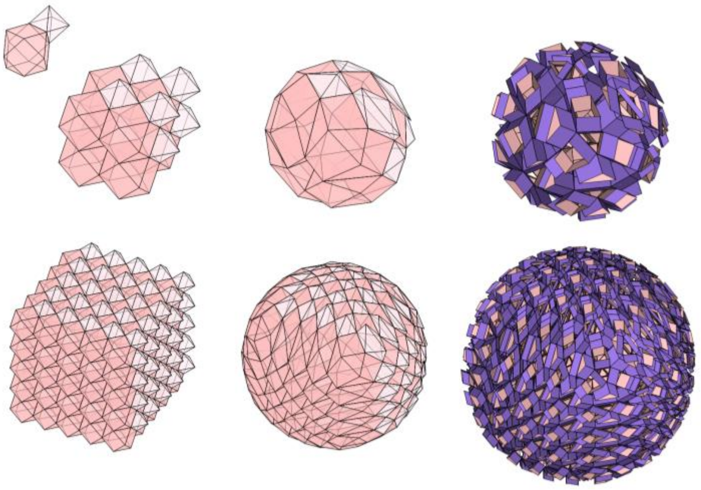

#8 octahedra and truncated cubes (1:1)

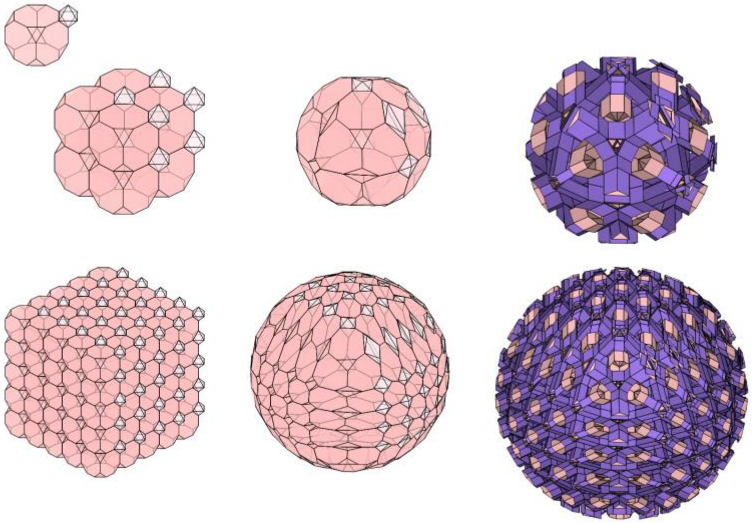

#9 cuboctahedra, rhombicuboctahedra and cubes (1:1:3)

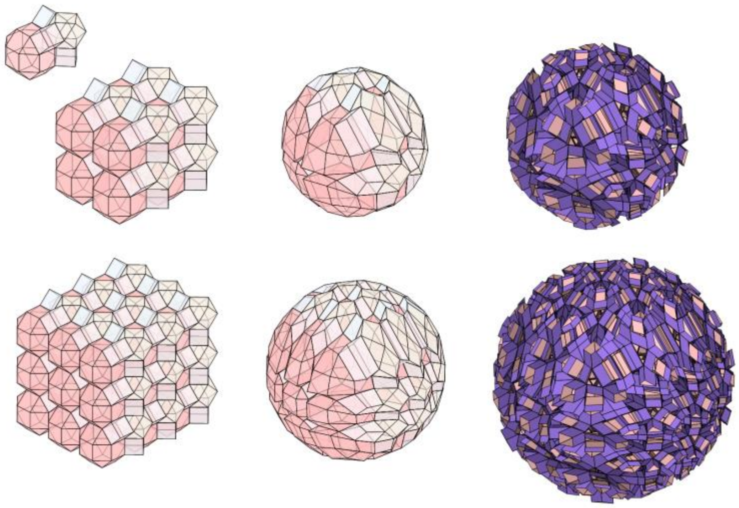

#10 cuboctahedra, truncated tetrahedra and truncated octahedra (1:1:2)

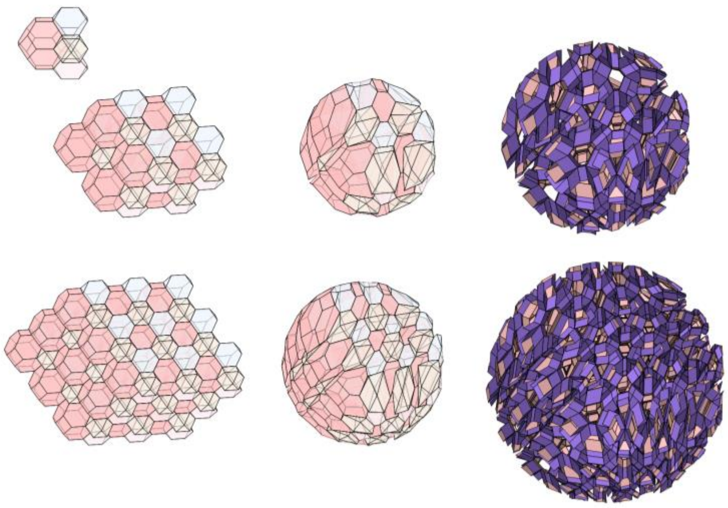

#11 Triangular prisms (2 of them comprise a unit)

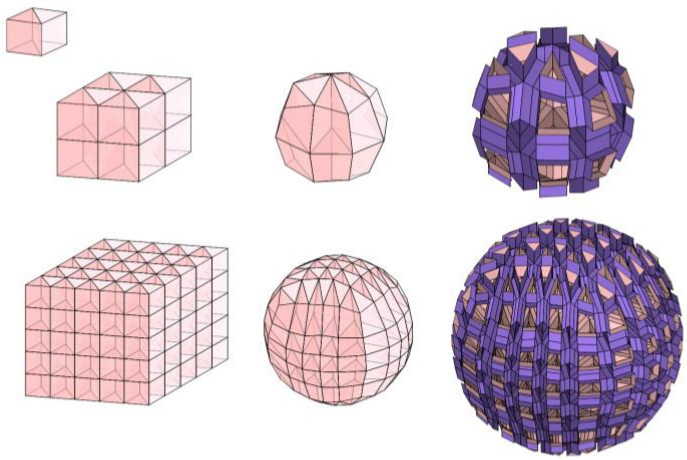

12 Triangular prisms (4 comprise a unit)

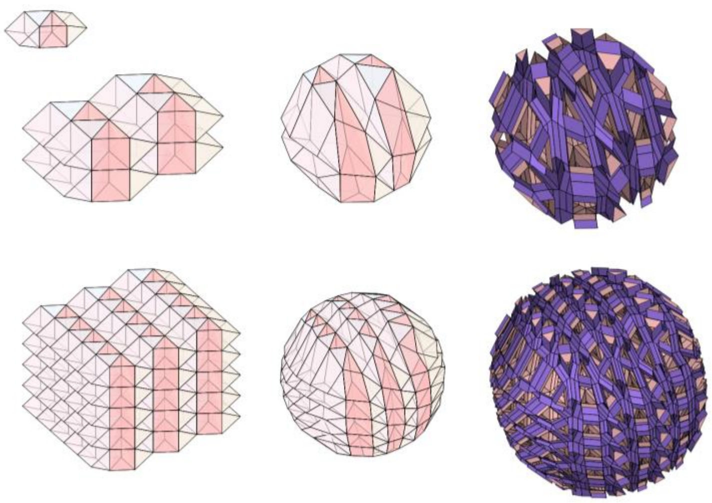

#13 triangular prisms and cubes (2:1)

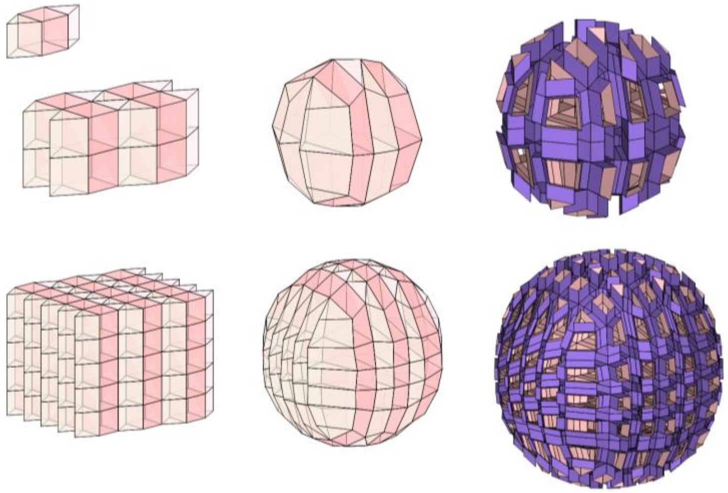

#14 triangular prisms and cubes (2:1)

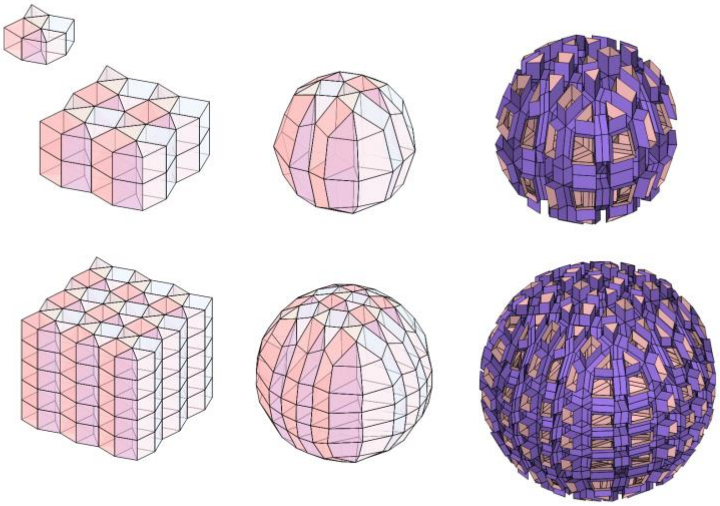

#15 triangular prisms and cubes (4:2)

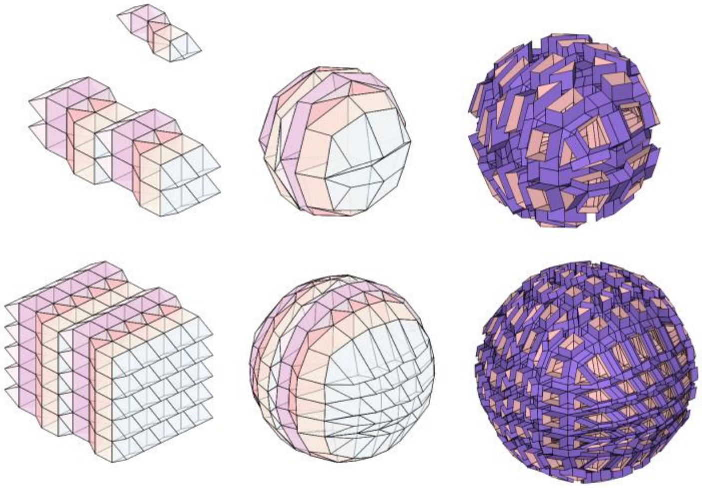

#16 triangular prims, cubes and hexagonal prisms (2:3:1)

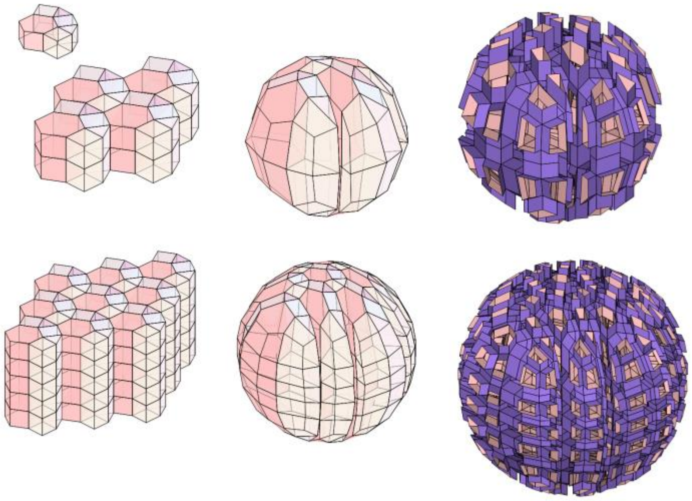

#17 triangular prisms and hexagonal prisms (8:1)

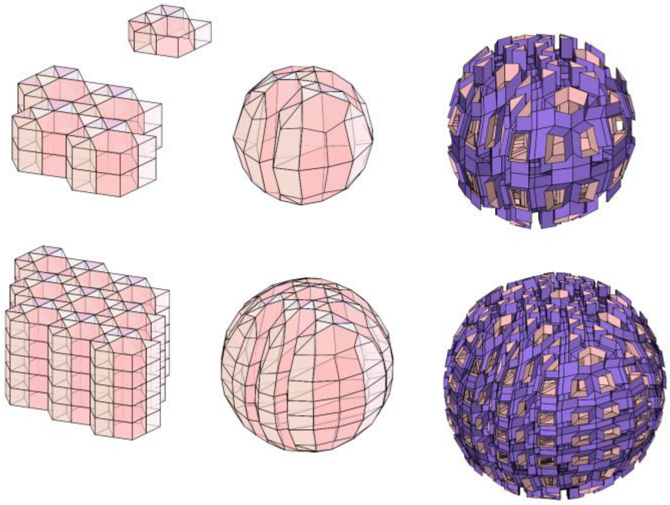

#18 triangular prisms and hexagonal prisms (2:1)

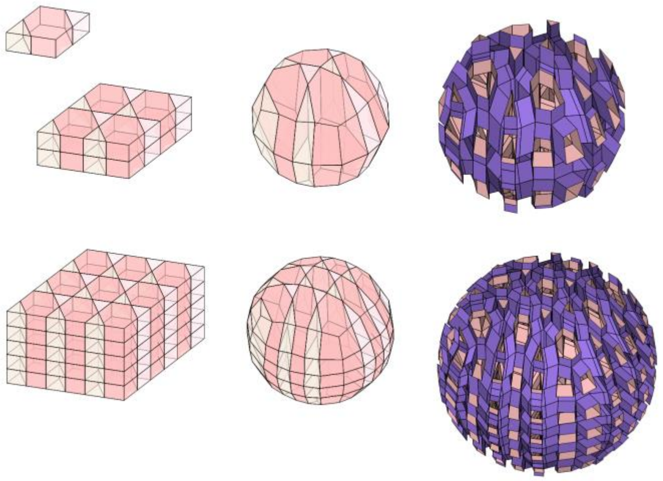

#19   triangular prisms and dodecagonal prisms (2:1)

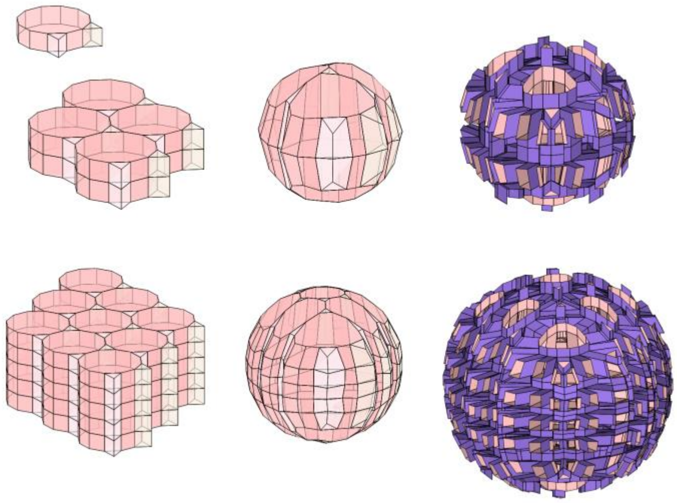

#20   rhombicuboctahedra, truncated cubes, cubes and octagonal prisms (1:1:3:3)

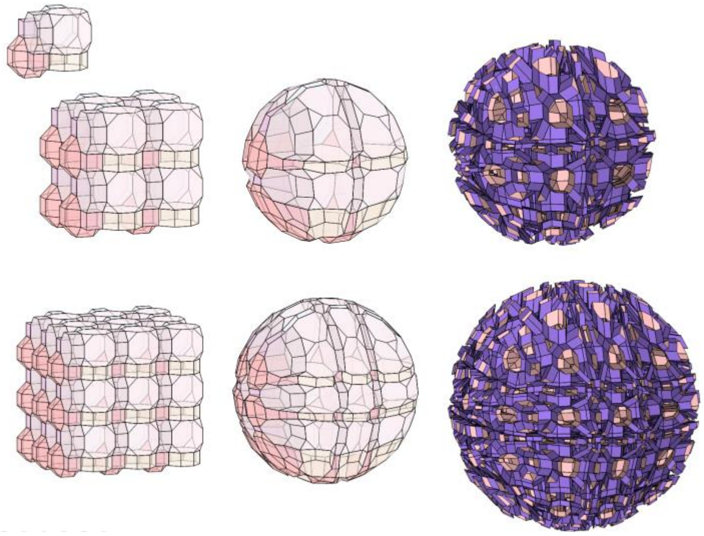

#21   truncated tetrahedra, truncated cubes and truncated cuboctahedra (2:1:1)

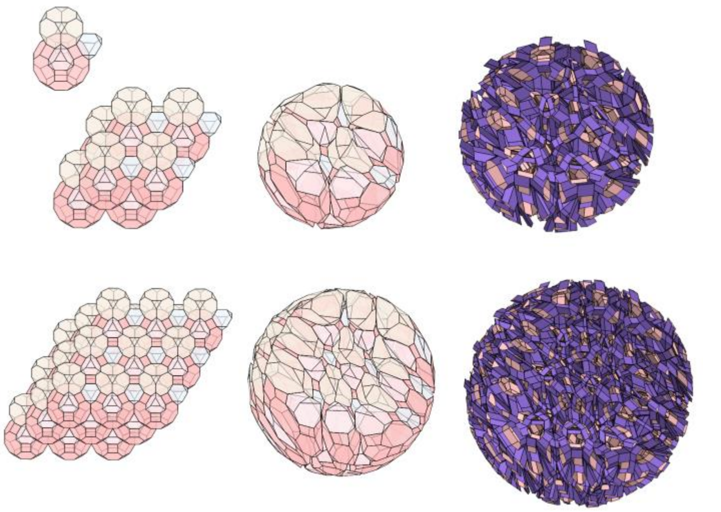

#22 cubes

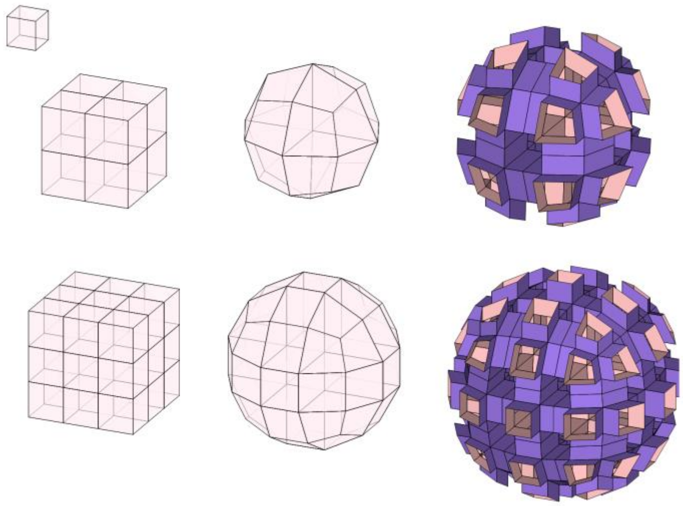

#23 cubes, hexagonal prisms and dodecagonal prisms (3:2:1)

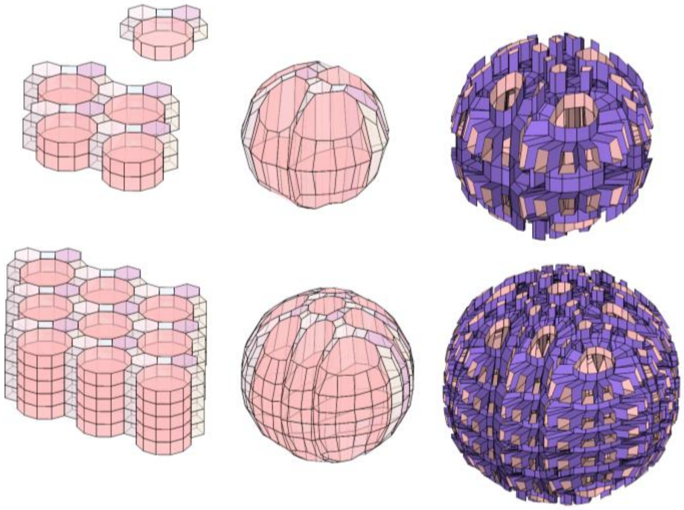

#24 cubes and hexagonal prisms (1:1)

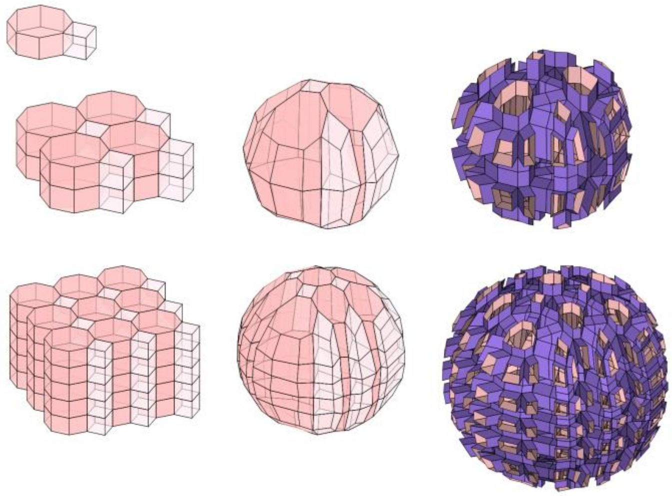

#25 cubes, truncated octahedra and truncated cuboctahedra (3:1:1)

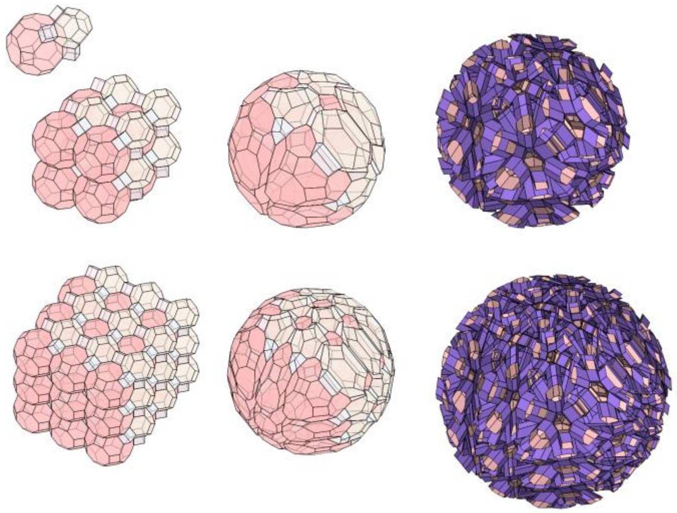

#26 hexagonal prisms

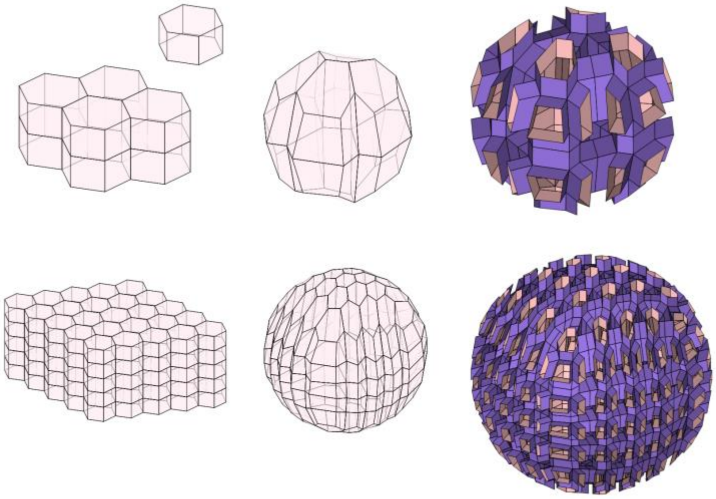

#27 octagonal prisms and truncated cuboctahedra (3:1)

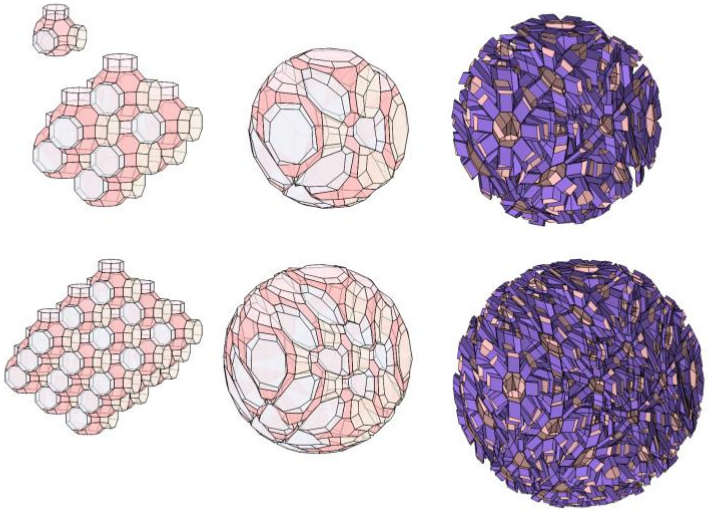

#28 truncated octahedra

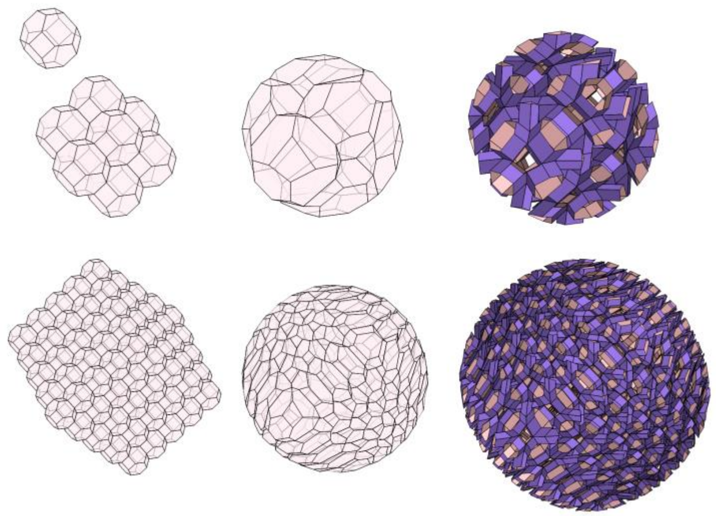

Supplementary Figure 3. Nonuniformly tessellated sphere structures constructed by geometric reconstruction of 28-unit cells: # 1 tetrahedra and octahedra (number of units 2:1); #2 tetrahedra and octahedra (number of units 4:2); #3 tetrahedra, octahedra, and triangular prisms (number of units 2:1:2); #4 tetrahedra, octahedra, and triangular prisms (number of units 4:2:4); #5 tetrahedra, rhombicuboctahedra, and cubes (number of units 2:1:1); #6 tetrahedra and truncated tetrahedra (number of units 2:2); #7 octahedra and cuboctahedra (number of units 1:1); #8 octahedra and truncated cubes (number of units 1:1); #9 cuboctahedra, rhombicuboctahedra and cubes (number of units 1:1:3); #10 cuboctahedra, truncated tetrahedra and truncated octahedra (number of units 1:1:2); #11 Triangular prisms (2 of them comprise a unit); # 12 Triangular prisms (4 comprise a unit); #13 triangular prisms and cubes (number of units 2:1); #14 triangular prisms and cubes (number of units 2:1); #15 triangular prisms and cubes (number of units 4:2); #16 triangular prims, cubes and hexagonal prisms (number of units 2:3:1); #17 triangular prisms and hexagonal prisms (number of units 8:1); #18 triangular prisms and hexagonal prisms (number of units 2:1); #19 triangular prisms and dodecagonal prisms (number of units 2:1); #20 rhombicuboctahedra, truncated cubes, cubes and octagonal prisms (number of units 1:1:3:3); #21 truncated tetrahedra, truncated cubes and truncated cuboctahedra (number of units 2:1:1); #22 cubes; #23 cubes, hexagonal prisms and dodecagonal prisms (number of units 3:2:1); #24 cubes and hexagonal prisms (number of units 1:1); #25 cubes, truncated octahedra and truncated cuboctahedra (number of units 3:1:1); #26 hexagonal prisms; #27 octagonal prisms and truncated cuboctahedra (number of units 3:1); #28 truncated octahedra.

Supplementary Note 2: Inverse design of reconfigurability

After the geometric reconstruction, the modules constructed from the shrunken polyhedrons generally produce immobility due to the irregular polygon tubes; the spatially connected irregular polygon tubes produce immobility. Some modules consist of a combination of regular and irregular polygon tubes, partially producing mobility. However, the mobility of the flexible modules is damaged when connected with adjacent rigid modules, as illustrated in Supplementary Figure 4.

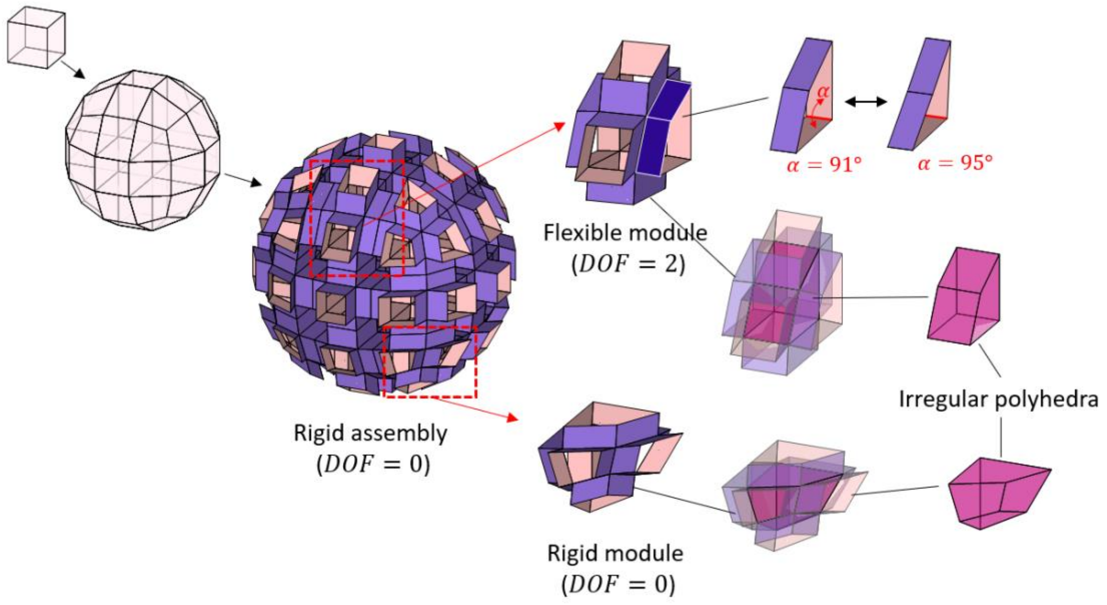

Supplementary Figure 4. Nonperiodic tessellation of a cubic unit cell producing different DOFs between the assembly and its modules. Rigid modules lock their mobility. Flexible modules consisting of partially regular and irregular polygon tubes have a limited range of motion.

Geometric modification with varying polygon tubes

Foldability conditions

The foldability constraints in Equations (4) and (5) in the main text, $\mathbf{I}_t \cdot \mathbf{d}_t = \mathbf{0}$ and $\mathbf{J}_q \cdot \boldsymbol{\theta}_q = \mathbf{0}$, vary depending on the shape of the polygons where prismatic tubes are extruded. Supplementary Figure 5 presents examples of the form of the foldability constraints for varying polygons. We focus on the tubes with parallel hinges to explain the form of $\mathbf{I}_t \cdot \mathbf{d}_t = \mathbf{0}$ in these examples; however, the principles of applying constraints are the same for the tubes with intersecting hinges, where $\mathbf{J}_q \cdot \boldsymbol{\theta}_q = \mathbf{0}$.

For instance, in the case in Supplementary Figure 5a.1 with the strictest constraints on foldability, we have

$$\mathbf{I}_t \cdot \mathbf{d}_t = \begin{bmatrix} 1 & -1 & 0 & 0 \\ 0 & 1 & -1 & 0 \\ 0 & 0 & 1 & -1 \end{bmatrix} \cdot \begin{bmatrix} d_{1_t} \\ d_{2_t} \\ d_{3_t} \\ d_{4_t} \end{bmatrix} = \begin{bmatrix} 0 \\ 0 \\ 0 \\ 0 \end{bmatrix}, \tag{1}$$

The quadrilateral tubes satisfying these constraints have a rhombic cross-section, reaching its maximum

sole motion for flat-foldability along any folding path.

For the tube in Supplementary Figure 5b.1 with an odd-number face in a pentagon shape,

$$\mathbf{I}_t \cdot \mathbf{d}_t = \begin{bmatrix} 1 & 0 & -1 & -1 & 0 \\ 0 & 1 & 0 & 0 & -1 \end{bmatrix} \cdot \begin{bmatrix} d_{1_t} \\ d_{2_t} \\ d_{3_t} \\ d_{4_t} \\ d_{5_t} \end{bmatrix} = \begin{bmatrix} 0 \\ 0 \\ 0 \\ 0 \\ 0 \end{bmatrix}, \quad (2)$$

The pentagonal tube reaches its maximum sole motion for flat-foldability along two possible folding paths.

In a single tube, a higher number of foldability constraints increase the range of motion. In the example where $f = 4$, the quadrilateral tube with a higher number of constraints is flat-foldable along any path, as shown in Supplementary Figures 5a.1 and a.2. However, the tube with one constraint can only be flat-folded along one path, as shown in Supplementary Figure 5a.3.

In this study, we applied the strictest foldability constraints possible, with the maximum number of constraints (r_t or r_q) for each tube — the cases of Supplementary Figure 5a.1, b.1, and c.1. However, when the targeting shape is complicated, it may be challenging for the algorithm to find a solution while simultaneously satisfying the maximum foldability of the tubes and other constraints during geometric modification, e.g., persevering the targeting shape and enforcing planar plates, as described in Equations (3) and (6) of the main text. In this context, we apply a weaker foldability constraint such as that in Supplementary Figures 5a.2, a.3, b.2, c.2, and c.3.

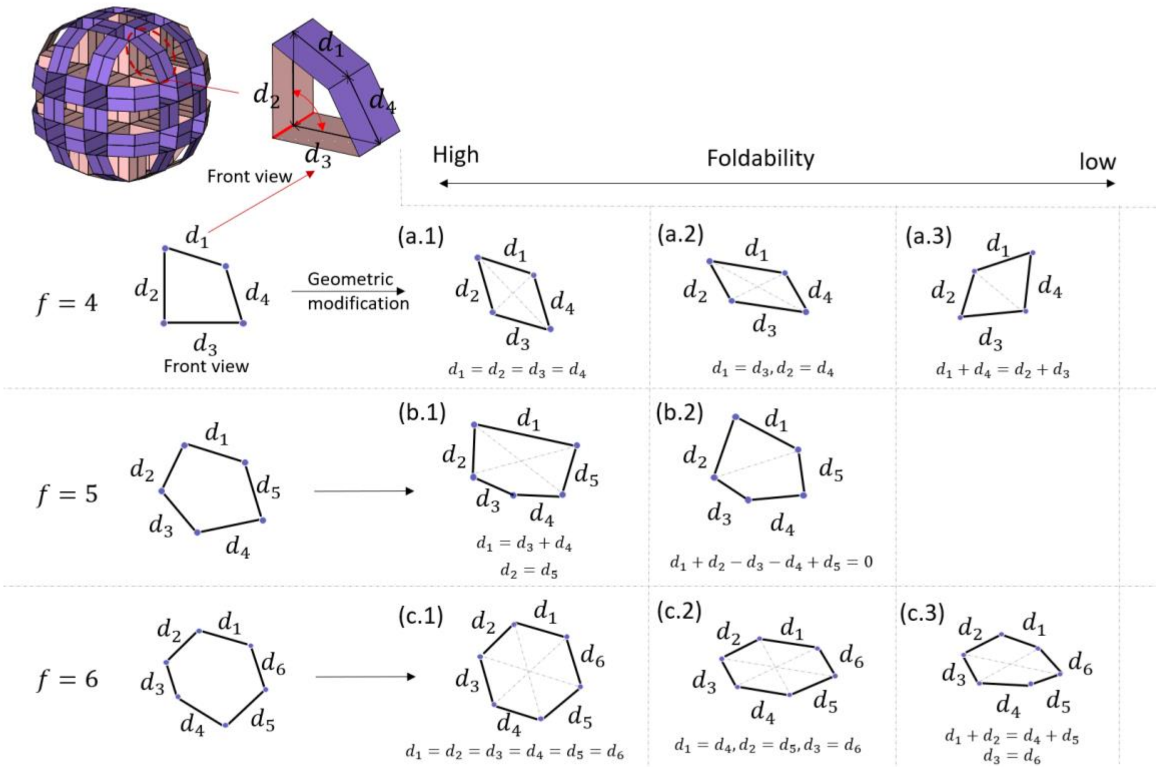

Supplementary Figure 5. Schematics of case-dependent foldability constraints: (a) the quadrilateral tubes (f=4) are viewed as a linkage from the perspective perpendicular to the hinges; (a.1) modified tube with the strictest foldability conditions, i.e., $d_1 = d_2 = d_3 = d_4$ (the folding paths are shown as the dashed lines connecting hinges pairs); (a.2) medium strict foldability constraints; and (a.3) loose foldability constraints; (b) modified tube in a pentagon shape with $f = 5$; (b.1) the modified tube can be folded flat along the two dashed lines; (b.2) the modified tube with only one folding path; (c) cases with different

foldability constraints in the hexagonal tube with $f = 6$. In this study, the tubes extruded from the triangular faces could be exempted from geometrical modification due to their immobility.

Implementation

Note that the prismatic architected structures produced by the geometric reconstruction serve as an initial guess for the design of reconfigurable structures. As a preprocess of reconfigurability, we proceed with a geometric modification by solving the optimization problem in Equations (3) to (6) in the main text using the optimization tool *fmincon* in MATLAB.

The objective function, foldability, and the planar-face constraints in Equations (3)–(6) in the main text are functions of nodal position vectors $(\mathbf{v}_1, \mathbf{v}_2, \mathbf{v}_3, \dots, \mathbf{v}_N)$ of the architected materials with full nodes N. We also specify gradient information for both the objective function and constraints using options of *SpecifyObjectiveGradient* and *SpecifyConstraintGradient* in *fmincon.*

The structural integrity of topological reconstruction

We apply the constraints on the graph structure in Equations (9) and (10) in the main text, $-C_i + 2 \leq 0$ and $C_p - 1 = 0$, where C_i is the number of connections on the i-th node. $C_p(\mathbf{x})$ is the number of connected graph components that ensure the structural integrity of the assembly of origami modules. Specifically, Equation (9) avoids local reconfigurability by excluding solely connected origami modules in the assembly of Supplementary Figure 6a; every module at least connects two adjacent ones. Constraint $C_p(\mathbf{x}) - 1 = 0$ on the graph structure avoids disconnection and biconnected topology in the origami assembly, as demonstrated in Supplementary Figures 6b and S6c. $C_p(\mathbf{x})$ can be calculated using the built-in function *conncomp* in MATLAB.

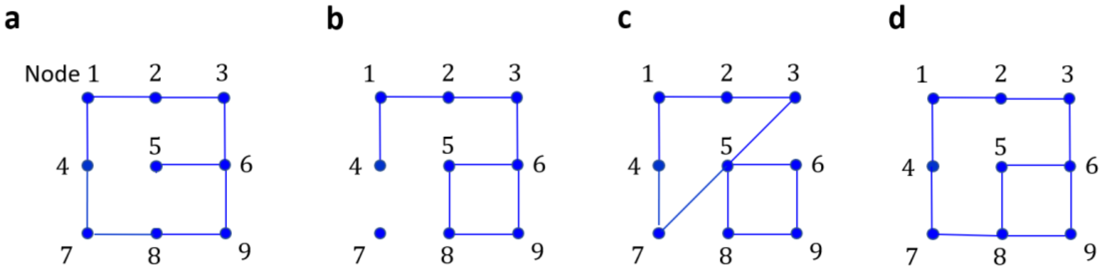

Supplementary Figure 6. Examples of topology (graph): (a) a disqualified topology due to the solely connected 5-th node; (b) a disqualified topology due to the disconnected 7-th node; (c) a disqualified topology due to the biconnected component, where $C_p = 2$;(d) a qualified graph.

Validation of top-down approach with 2D motion structures

To validate our inverse design with the kinematic mobility analysis (bottom-up approach), we present simple assemblies of 2D bar mechanisms. While removing links (bars) from a 3×3 linkage in Supplementary Figures 7a and 7b, we obtain mobility using Kutzbach's modification of Gruebler's mobility equation:

$$M = 3(L - 1) - 2(J_1 + J_m), \qquad (3)$$

where M is the mobility, L is the number of links, J_1 is the number of full joints, and J_m is the number of multiple joints counting as one less than the number of links joined at the point. Note that the nodes and edges in Supplementary Figures 7a and 7b represent hinges and binary links, respectively.

We compare the kinematic analysis with our inverse design via the topological reconstruction. Similar to Equations (7)–(10) in the main text, we formulate the design problem for target mobility $\bar{N}_{dof}$:

$$\min \left(N_{dof}(\mathbf{x}) - \bar{N}_{dof}\right)^2 \tag{4}$$

$$s.t. \qquad x_i \in [0,1] \qquad \text{for } i = 1,2,3,\ldots,N_e \tag{5}$$

$$-C_j(\mathbf{x}) + 2 \leq 0, \qquad \text{for } j = 1,2,3,\ldots,N_v \tag{6}$$

$$C_p(\mathbf{x}) - 1 = 0, \tag{7}$$

where the binary design parameter x_i represents the existence of the x_i-th link, with 0 representing the links removed. N_e and N_v are the total number of links and hinges before removal, respectively. $N_{dof}(\mathbf{x})$ is the mobility for a combination of $\mathbf{x}$. The constraints in Supplementary Equations (6) and (7) control the structural integrity of the assembly, where $C_i(\mathbf{x})$ is the number of connections on the i-th node and $C_p(\mathbf{x})$ is the number of connected graph components.

As shown in Supplementary Figure 7c, our inverse design validates the analytical solution of kinematics. Notably, our top-down approach computationally explores the entire design space; our inverse design provides multiple solutions for a targeting n_{dof}, as shown in Supplementary Figures 7c–7e.

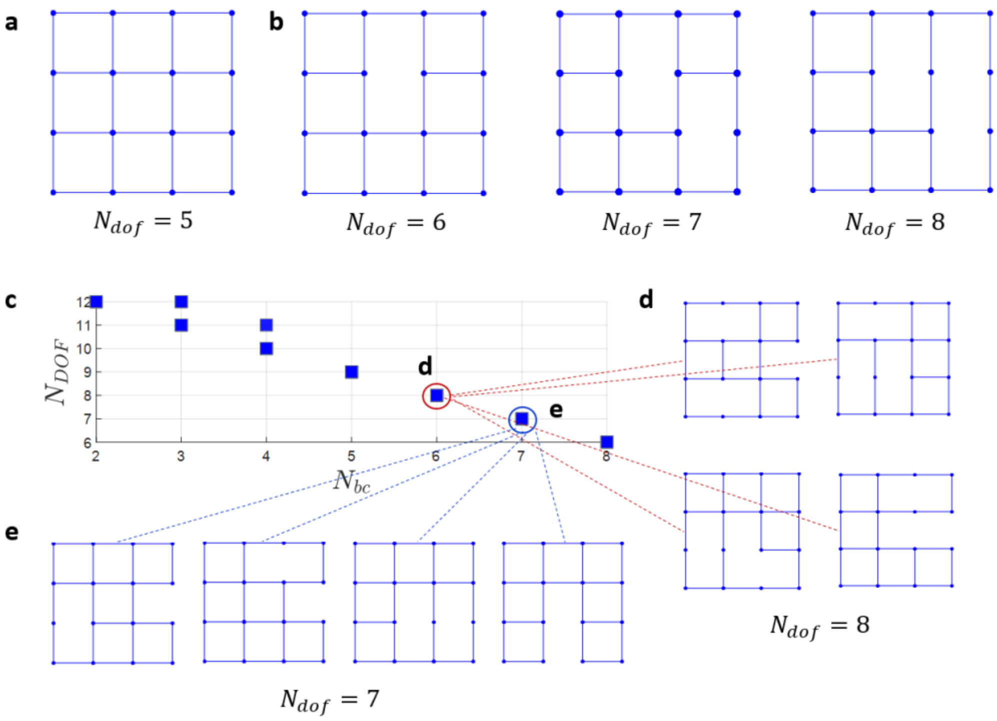

Supplementary Figure 7. Validation of the inverse design with the kinematic mobility analysis: (a) a fully connected bar-linkage mechanism ($N_{dof} = 5$) in 3 x 3 size; (b) linkages and their mobility calculated using Gruebler's mobility equation; (c) a scatter plot showing 30 designs identified using the topological reconstruction algorithm; each point represents one or several distinct designs. For example, there are four different designs for (d) $N_{dof} = 8$ and (e) $N_{dof} = 7$.

Supplementary Note 3: Mobility analysis and simulation of transformation

To identify the DOFs of reconfigurable modular origami comprising rigid faces and flexible hinges, we calculate the number of free variables associated with the linearized constraint matrix[1]. We triangulate each face of the structure to facilitate the description of geometric constraints, for which an additional diagonal edge is generated on the face, as shown in Supplementary Figure 8a. We describe the length constraint on two vertices on the i-th edge as $\left(\mathbf{v}_{a_i} - \mathbf{v}_{b_i}\right) \cdot \left(\mathbf{v}_{a_i} - \mathbf{v}_{b_i}\right) = L_i^2$, for $i = 1,2,3, \dots, E$, where $\mathbf{v}_{a_i}$ and $\mathbf{v}_{b_i}$ are the position vectors of the two vertices, L_i is the length of the i-th edge, and E is the number of total edges. Next, we prescribe the plane constraints of flat faces such that all the vertices on the j-th face remain planar during the transformation, i.e., $(\mathbf{v}_{4_j} - \mathbf{v}_{2_j}) \cdot \left[(\mathbf{v}_{1_j} - \mathbf{v}_{2_j}) \times (\mathbf{v}_{3_j} - \mathbf{v}_{2_j})\right] = 0$ for $j = 1,2,3, \dots, F$, where F is the total number of faces. After linearizing the above two constraints and assembling them into a matrix $\mathbf{J}_v$, we obtain $\mathbf{J}_v \cdot d\mathbf{v} = \mathbf{0}$, where $d\mathbf{v}$ is a vector indicating the infinitesimal displacement of every vertex. Finally, the number of DOFs is obtained as $3 \cdot n_v - \mathrm{rank}(\mathbf{J}_v) - 6$, where n_v is the total number of vertices in the structure and the rank of $\mathbf{J}_v$ indicates the number of independent constraints.

The transformation process of a multi-DOF reconfigurable structure can be simulated by folding the dihedral angles of independent hinges. To find such independent hinges, we combine $d\boldsymbol{\phi} = \mathbf{J}_h \cdot d\mathbf{v}$, where $d\boldsymbol{\phi}$ is the infinitesimal change in all the hinge angles, with the equation $\mathbf{J}_v \cdot d\mathbf{v} = \mathbf{0}$, leading to $\mathbf{J} \cdot \begin{bmatrix} d\mathbf{v} \\ d\boldsymbol{\phi} \end{bmatrix} = \mathbf{0}$. Here, we have $\mathbf{J} = \begin{bmatrix} \mathbf{J}_v & \mathbf{0} \\ \mathbf{J}_h & -\mathbf{1} \end{bmatrix}$, as mentioned in the main text. For $d\boldsymbol{\phi} = \mathbf{J}_h \cdot d\mathbf{v}$, the Jacobian matrix $\mathbf{J}_h$ has the following entries:

$$J_{h[r,3\cdot(n-1)+j]} = \frac{\partial \phi_r}{\partial v_{j,n}}, \tag{8}$$

where $j = 1,2,\text{and } 3$. $dv_{j,n}$ is the displacement of the n-th vertex along the j-th axis of a Cartesian coordinate, $r = 1, \dots T$. T is the total number of selected hinges, and $n = 1,2,3 \dots, N$. N is the total number of vertices in the structure. A detailed derivation of $\mathbf{J}_h$ can be found elsewhere[2].

Next, we find the free column in the reduced row echelon form of $\mathbf{J}$. The free column corresponds to free variables in $\begin{bmatrix} d\mathbf{v} \\ d\boldsymbol{\phi} \end{bmatrix}$. All the free variables can be found in $d\boldsymbol{\phi}$, i.e., the independent angles.

We obtain the transformed configurations of the reconfigurable architected materials using a numerical iterative method that applies a projection matrix to reduce numerical errors[3]. In each iteration, the input is a slight increase $\begin{bmatrix} \mathbf{0} \\ d\boldsymbol{\phi}_0 \end{bmatrix}$ in the actuating angles, and the output is a displacement vector $\begin{bmatrix} d\mathbf{v} \\ d\boldsymbol{\phi} \end{bmatrix}$ while satisfying the kinematics constraints. Between the input and output, there is

$$\begin{bmatrix} d\mathbf{v} \\ d\boldsymbol{\phi} \end{bmatrix} = -\mathbf{J}^{+}\mathbf{r} + [\mathbf{I} - \mathbf{J}^{+}\mathbf{J}] \begin{bmatrix} \mathbf{0} \\ d\boldsymbol{\phi}_0 \end{bmatrix}, \tag{9}$$

where $[\mathbf{I} - \mathbf{J}^{+}\mathbf{J}]$ is the projection matrix, $\mathbf{J}^{+}$ is the pseudo-inverse of $\mathbf{J}$, and $\mathbf{I}$ is a unit matrix. The term $-\mathbf{J}^{+}\mathbf{r}$ in Supplementary Equation (9) reduces the numerical error in each iteration with a residual vector $\mathbf{r} = \left[\mathrm{r}_{e_1}, \mathrm{r}_{e_2}, \dots, \mathrm{r}_{e_E}, \mathrm{r}_{f_1}, \mathrm{r}_{f_2}, \dots, \mathrm{r}_{f_F}\right]^T$, where $\mathrm{r}_{e_i} = \left(\mathbf{v}_{a_i} - \mathbf{v}_{b_i}\right) \cdot \left(\mathbf{v}_{a_i} - \mathbf{v}_{b_i}\right) - L_i^2$ is the residual of the constraint of the i-th edge and $\mathrm{r}_{f_j} = (\mathbf{v}_{a_j} - \mathbf{v}_{2_j}) \cdot \left[(\mathbf{v}_{1_j} - \mathbf{v}_{2_j}) \times (\mathbf{v}_{3_j} - \mathbf{v}_{2_j})\right]$ is the residual of the constraint for the j-th face. $\mathbf{r} = [\mathbf{0}]$ in the first iteration.

To avoid overlapping of the faces during the transformation, we limit the range of dihedral angles of every hinge to $0 \leq \phi \leq \pi$, where

$$\phi = \pi - \tan^{-1}\left(\mathbf{p} \cdot \frac{(\mathbf{n}_a \times \mathbf{n}_b)}{\mathbf{n}_a \cdot \mathbf{n}_b}\right). \tag{10}$$

Here, the normal vectors on the faces are $\mathbf{n}_a = \frac{\mathbf{p}\times\mathbf{q}_b}{|\mathbf{p}|\cdot|\mathbf{q}_b|}$ and $\mathbf{n}_b = \frac{\mathbf{q}_b\times\mathbf{p}}{|\mathbf{q}_b|\cdot|\mathbf{p}|}$, as illustrated in Supplementary Figure 8b.

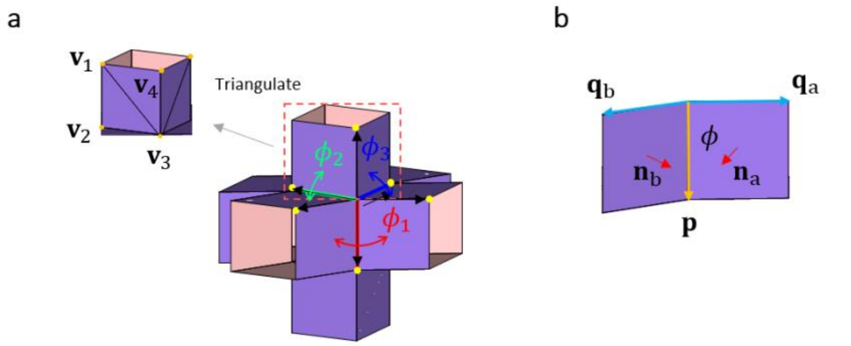

Supplementary Figure 8. An example to identify rigid faces and flexible hinges; (a) a schematic of the triangulated face of a modular origami module; (b) the dihedral angle with the associated vectors.

Supplementary Note 4: Fabrication of physical models

Rigid structures

The geometry reconstruction using the volumetric mapping and shrinkage in the main text provides stiff and immobile structures suitable for lightweight structural applications. We prototype a rigid model using multi-material inkjet 3D printing (MultiJet, ProJet MJP 5600, 3D systems). To generate an input file for 3D printing, we write a MATLAB script converting a thin-walled model (Supplementary Figure 9a) into one with a prescribed wall thickness (Supplementary Figure 9b). For the multiJet printing, the thicknesses of the plate t_p and hinges t_h can be close ($0.7t_p \leq t_h \leq t_p$), as shown in Supplementary Figure 9c. We assign VisiJet CR-CL (E~1.3GPa) to the faces and VisiJet CE-BK (E~$0.3\ MPa$) to the hinges of the model. Supplementary Figure 9d shows a printed prototype.

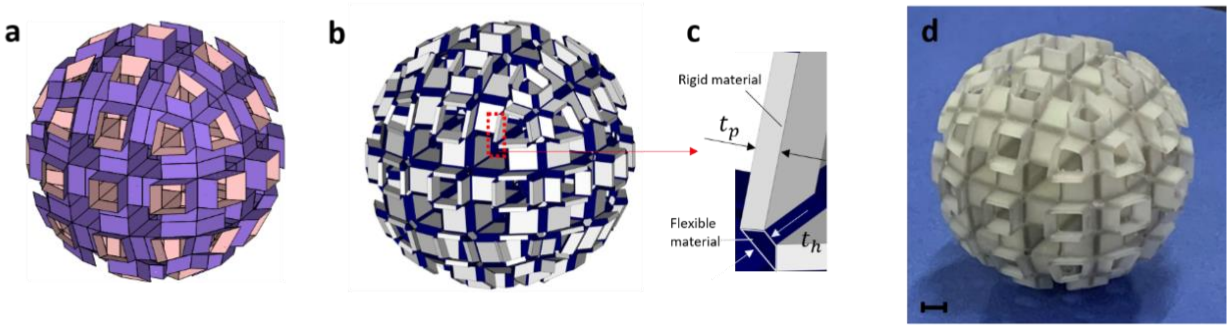

Supplementary Figure 9. A rigid origami structure: (a) a spherical prismatic architected material with $3 \times 3 \times 3$ modules after the geometric reconstruction by the spatially gradient tessellation of a cubic reference unit; (b) meshed file of (a) in SOLIDWORKS; (c) a zoomed-in view of a hinge and face; (d) prototype prepared using multiJet printing. Scale bar, 1cm.

Reconfigurable structures

The geometric modification and topological reconstruction in the main text can build reconfigurable structures. We fabricate reconfigurable prototypes using i) assembly of patterned paperboards and ii) additive manufacturing by stereolithography (SLA) using Form 3 (Formlabs). For the assembly of a thin-walled model in Supplementary Figure 10a, we generate a papercutting path on paperboards (Supplementary Figure 10b) using an in-house pattern cutting machine, followed by connecting hinges with transparent tapes, as shown in Supplementary Figure 10c. Seeking fully automatic manufacturing, we utilize additive manufacturing with a single material. We set $0.05t_p \leq t_h \leq 0.5t_p$ and assign a single soft material (Flexible 80A with E~2.3 MPa) for the entire model, as shown in Supplementary Figures 10d and 10e. Supplementary Figure 10f shows a printed reconfigurable prototype.

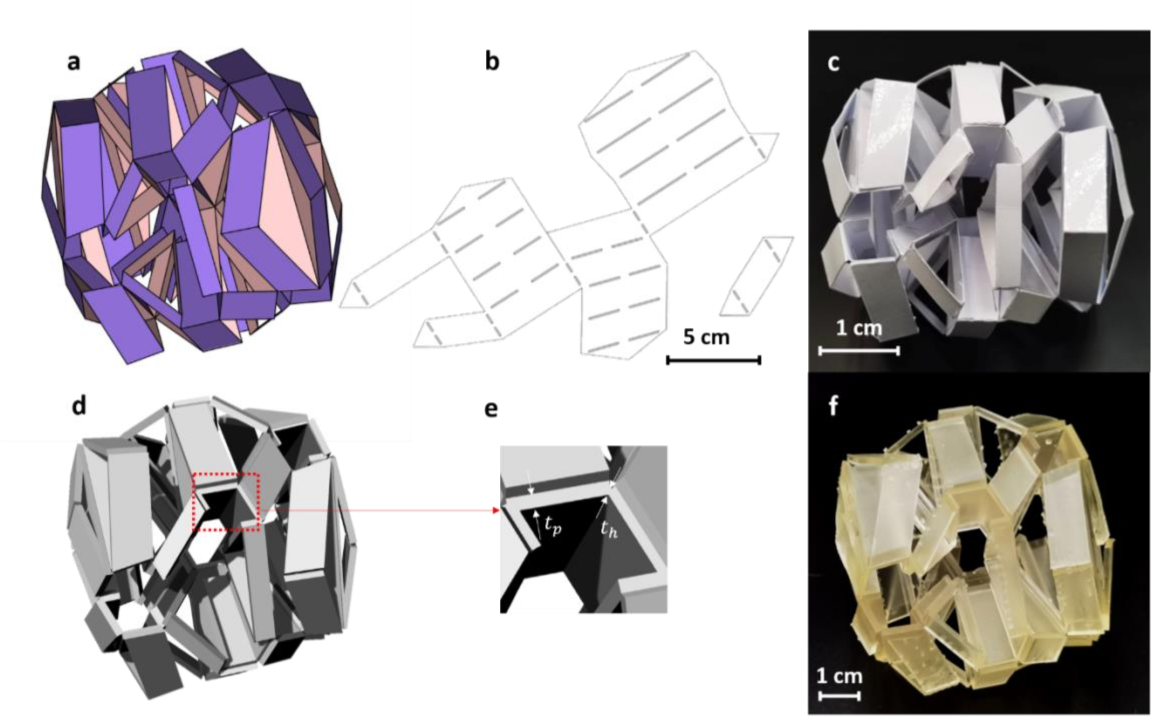

Supplementary Figure 10. A reconfigurable modular origami structure: (a) a spherical prismatic modular origami structure with a spatially gradient tessellation of a tetrahedron reference unit; (b) the papercutting path of a selected module from (a) in SOLIDWORKS; (c) a paperboard prototype of the model (a); (d) a meshed file of (a) in SOLIDWORKS; (e) a zoomed-in view of a hinge and a face; (f) a prototype prepared by SLA printing.

Magnetic actuation

To demonstrate a reconfigurable structure (Supplementary Figure 11a) remotely controllable, we printed a hyperboloid prismatic modular origami using the topological reconstruction of tetrahedron modules and selectively embedded six permanent magnets (NdFeB) into triangular tubes, as shown in Supplementary Figures 11c and 11d. We used ethyl vinyl acetate (EVA) hot glue to bond the magnets to the printed prototypes; the location of the magnets and their remnant flux orientation are shown in Supplementary Figures 11c and 11d.

We apply a rotational uniform magnetic field to actuate the 3D printed prototype. The uniform magnetic field is generated by a Halbach array composed of a circumferential array of permanent magnets, where the cylindrical magnetic space has diameter $D\,(=104mm)$ and height $h\,(=26mm)$, as shown in Supplementary Figure 11b. A uniform magnetic field is applied in the radial direction of the cylindrical hole with a magnitude of 80 mT.

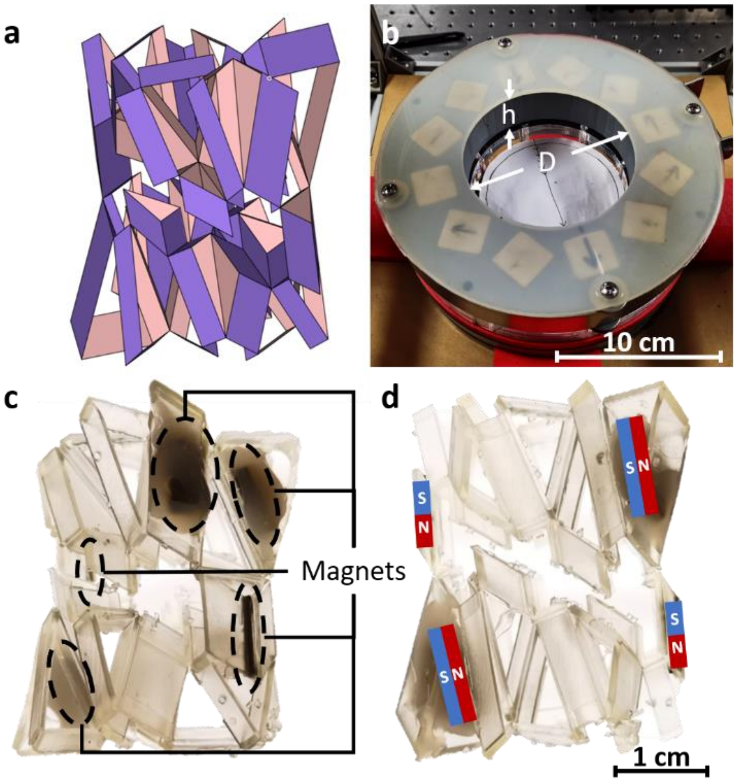

Supplementary Figure 11. A magnetically triggered reconfigurable structure: (a) hyperboloid reconfigurable modular origami structure with a spatially gradient tessellation of a tetrahedron reference unit followed by geometric modification and topological reconstruction; (b) the Halbach array provides a rotational uniform magnetic field; (c) locations of permanent magnets embedded in the prototype; (d) remanent flux orientation of permanent magnets in the hyperboloid prototype.

Validation of motion with experiment

To validate our numerical algorithm of inverse design of reconfigurability, we fabricated one reconfigurable structure — the spherical structure with tetrahedron and octahedron modules of Figure 3b in the main text. Note that the reconfigurable structure has 10 DOFs after the topological reconstruction, as shown in Figure 3a7 of the main text. We used a 3D scanner (Einscan Pro) to capture the transformed shapes of an SLA 3D printed prototype. We compared the 10 dihedral angles of the reconfigurable structure during transformation obtained by numerical simulation and 3D scanned measurement, as shown in Supplementary Figure 12.

Our model reasonably matches the experiment. The measured configurations were close to the simulated ones, as shown in Supplementary Figures 12a–12d. The measurement shows an average mismatch of $\sim 3° \leq \frac{1}{N}\sum|\Delta\phi| \leq 13°$ depending on the configurations and angles measured. Note that it is challenging to match our model with the experiment due to the comprehensive and accumulated errors such as calibration errors by the perspective 3D scanning view, hidden parts of interior connections during 3D scanning, dimensional errors during 3D printing, and so on.

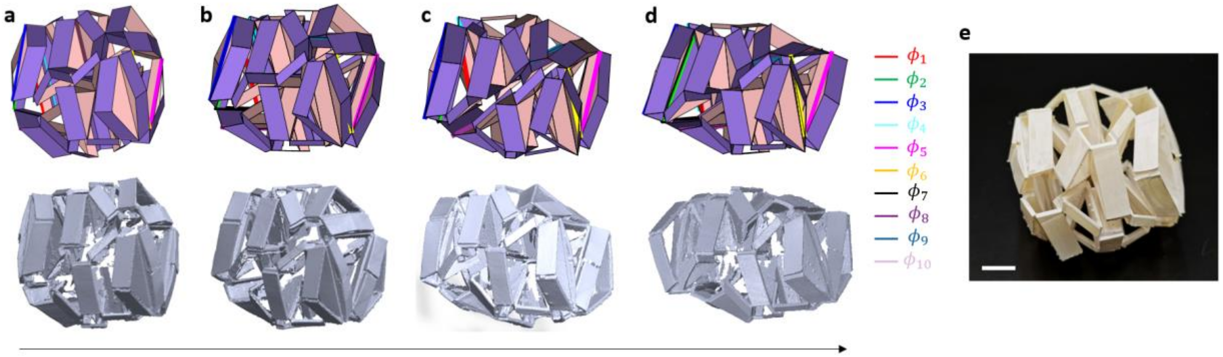

f

	ϕ_1	ϕ_2	ϕ_3	ϕ_4	ϕ_5	ϕ_6	ϕ_7	ϕ_8	ϕ_9	ϕ_{10}	$\frac{1}{N}\sum\lvert\Delta\phi\rvert$
Sim.(a)	73°	73°	107°	107°	71°	71°	73°	107°	71°	109°	3.34°
Exp.(a)	77.78°	75.70°	103.31°	101.03°	72.41°	76.47°	77.78°	108.92°	73.61°	109.05°	
Sim.(b)	80°	80°	94°	94°	84°	84°	65°	104°	77°	107°	5.16°
Exp.(b)	80.02°	79.91°	79.85°	84.68°	84.13°	83.10°	70.01°	110.89°	85.02°	114.07°	
Sim.(c)	108°	108°	79°	79°	98°	98°	35°	134°	93°	134°	8.36°
Exp.(c)	108.15°	103.15°	71.91°	78.32°	100.94°	92.85°	67.71°	123.83°	93.26°	114.31°	
Sim.(d)	124°	124°	67°	67°	111°	111°	16°	147°	103°	145°	13.69°
Exp.(d)	119.84°	121.96°	71.39°	63.00°	108.94°	106.52°	71.72°	117.23°	90.2°	127.5°	

Supplementary Figure 12. Quantitative comparison of transformations obtained from numerical simulations and experimental measurements using 3D scanning: (a)–(d) transformed configurations of a spherical architected structure with tetrahedron and octahedron modules; (e) a 3D-printed prototype for 3D scanning; (f) dihedral angles from the numerical model and experimental measurement; upper row: numerical model with 10 dihedral angles ϕ as DOFs; lower row: measurement by 3D scanning.

Supplementary References:

1 Laliberté, T. & Gosselin, C. Construction, mobility analysis and synthesis of polyhedra with articulated faces. *Journal of Mechanisms and Robotics* **6**, 011007 (2014).

2 Overvelde, J. T., Weaver, J. C., Hoberman, C. & Bertoldi, K. Rational design of reconfigurable prismatic architected materials. *Nature* **541**, 347-352 (2017).

3 Tachi, T. Simulation of rigid origami. *Origami* ***4***, 175-187 (2009).
