# Supplementary material for: Inverse design of 3D reconfigurable curvilinear modular origami structures using geometric and topological reconstructions
Source: Nat Commun. 2022 Dec 3;13:7474. doi: 10.1038/s41467-022-35224-2 (PMC9719498; doi:10.1038/s41467-022-35224-2)
Supplement: Supplementary file 3 — Description of Additional Supplementary Files [file 41467_2022_35224_MOESM3_ESM.pdf]

Description of Additional Supplementary Files

File Name: Supplementary Movie 1
Description: Geometric reconstruction by volumetric mapping and shrinkage

File Name: Supplementary Movie 2
Description: Topological reconstruction

File Name: Supplementary Movie 3
Description: Reconfigurability of a 3D modular origami constructed by the topological reconstruction

File Name: Supplementary Movie 4
Description: Reconfigurable 3D modular origamis with different target shapes – hyperboloid and cone

File Name: Supplementary Movie 5
Description: The effect of topology on reconfigurability
